# Supplementary material for: Comparative analysis of the ex vivo IFN-gamma responses to CD8+ T cell epitopes within allelic forms of PfAMA1 in subjects with natural exposure to malaria
Source: PLoS One. 2021 Sep 10;16(9):e0257219. doi: 10.1371/journal.pone.0257219 (PMC8432784; doi:10.1371/journal.pone.0257219)
Supplement: Attachment — Submitted filename: Reviewer comments for author.docx [file pone.0257219.s002.docx]

-

| **Reviewer’s comments for the author.** | | |
| --- | --- | --- |
| The study attempt to understand allelic polymorphisms in the malarial antigen apical membrane antigen 1 (AMA1) and the effect on *ex vivo* T cell-specific IFN-γ responses in a population with lifelong exposure to malaria.  The manuscript is well written.  The strength of the manuscript is that it speaks to the design of malaria vaccine candidate, a global need and very relevant to today’s malaria science.  The weakness of the manuscript is that the results are based on ONLY 7 candidate/individuals study. This sample size is very small and inferences based on such sample size is statistically challenging.  These said, there are a number of issues/concerns that I believe would further improve the manuscript if looked into. | | |
|  | **Background** |  |
| Line No | Extract from manuscript | Reviewer’s comment |
| Line 49 -51  51 | Malaria remains an economically important disease globally as it results in a huge loss in productivity amongst affected populations and requires significant financial inputs for control and prevention.  found mostly in Africa region | This statement should be ref  grammatical errors ? rephrase |
| 54 - 55 | available evidence points to the need for additional complementary tools to ensure disease elimination and eradication | Vague, what is the evidence? Why is there the need for these complementary tools |
|  | **Materials and Methods** |  |
| 100 -125 101 | This study therefore investigated the effect of allelic polymorphisms in MHC-restricted peptides from AMA1 on *ex-vivo* malaria parasite-specific IFN-γ responses in a population with history of | The authors should state the rationale for their selection of AMA1 considering there are other protective antigens in P. falciparum that have been confirmed to show huge allelic polymorphism |
| 116 | **Study Site, participants and sample collection** | A map of this location would add value. |
| 119 - 120 | periods of rainfall between March and November. | Were blood samples collected from the seven subjects during this period of March to November when malaria infection is known to be high in this region? The authors need to be specific here |
| 122 - 123 | participation in a previous study by the same group, in which participants’ HLA A and B types were determined, | Is this necessary since all participants were males |
| 125 126 | All seven subjects met the inclusion criteria and tested negative for malaria parasites by light microscopy. | Were the seven subjects randomly selected from different households in Legon community? This should also be stated together with the inclusion criteria to ensure representativeness in this community |
| 127 | Sixty ml (60 ml) of venous blood was collected per subject into heparinized tubes | Was the isolation of PBMC from the blood done on the same day of sample collection? If no, then the authors should state the procedure for preservation of the blood samples |
| 128 | previously described [23] | Briefly in one or two sentences summarized this procedure. |
| 135 | NetMHC algorithm | Provide the parameters of NetMHC used for 9-10mer peptide prediction |
| 137 | was aligned | The author should state the kind of alignment and the software used |
| 141 - 142 | have amino acid variability and, based on the multiple sequence alignments, were selected | Sentence structure needs revision |
| 145 | and following alignment with the other four allelic AMA1 sequences | What tool was used for this alignment?? |
| 146 - 147 | an additional 49 peptides with variability in at least one amino acid residue position relative to the predicted 3D7 consensus peptides were identified. | This speaks of the result of this alignment and as such should be in the result section |
| 149 - 151 | Thirty-five (35) of the 42 predicted 3D7 peptides were shared with one or more of the 4 other parasite strains, while the seven remaining peptides were unique to the 3D7 clone only | All these should be in the results section |
| 152 - 153 | All synthesized peptides had > 91 % purity and were originally lyophilized. Peptides were initially dissolved in DMSO and diluted with sterile plain RPMI-1640 to a stock concentration of 10 mg/ml. | Peptide synthesis does not fall under the sub-heading “**Predicted 3D7 class 1 epitopes and matching variant sequence synthesis”.** As such it should be under a new sub-heading and the procedure for synthesis should be stated. |
| 187 – 189 | For peptides within an allelic set, sfc/m were compared by either Student *t* test (for paired peptides) or by One-way ANOVA (for more than 2 peptides) as | Was this done manually or how?? Please give more information |
| **Results** | | |
| 221 | to PBMC stimulations by all peptides ranged from 0 to 66 sfc/m. | There is high variation in this from one participant to the next. I see it is mentioned in the discussion also, but what is the implication of that on the study and statistical analysis? |
|  | **Fig 1. Alignment of *P. falciparum* AMA1 sequences from the five parasite strains.** | figure 1- Figure resolution needs improvement |
| 321 | **Table 4: Outcome of subject 4 PBMC stimulation with allelic peptide sets** | Why is this table separate from Table 3? Combining both might improve presentation. |
| 365 | that did not elicit positive T cell responses. | Is the positive IFN-gamma associated with T-cell response. Please clarify |
| 371 -376 | Of the eight positive peptides whose corresponding alleles showed statistically significantly less responses, a total of 12 amino acid substitutions were present. Three substitutions occurred at each of amino acid positions 1 and 6. Substitutions at position 1 were from a polar to a positively charged residue (Table 1), from a negatively charged to a positively charged residue (Table 2) and from one non-polar residue to another (Table 3). At position 6, substitutions were from polar to a positively charged residue (Table 1) and two from non-polar to a polar residue (Tables 1 and 4). | Was a significant relationship observed between the polarity of amino acids and immune induction (In this case IFN-gamma and T-cell)? I think this area should have focus on whether the amino acid substitution result in changes of the peptide structure that could have resultant effect on its ability to induce immune response. |
| 384 - 385 | **Fig 2. Frequency of polymorphic residue distribution at each amino acid position within test peptides.** | A sharp figure is require. Figure resolution can be improved |
| This study only used 7 subjects, but the results showed variability between those 7. What is expected for larger sample sizes? Would the variability even out? What does this mean for the population at large | | |
|  | **Discussion** |  |
|  |  | Some questions I thought of during results were addressed here, which is good. |
| 424 - 425 | Three of the 18 positive peptides had IFN-γ responses above 100 sfc/m PBMCs while those of the other 15 positive peptides were between 10 and 100 sfc/m. | As mentioned in the results, the variation may be allowed but does this mean It has no significant effect on the analysis? |
| 450 - 451 | The predominant positions where changes that accounted for different responses occurred were at residues 1 and 6. | Have these residues been reported as being of interest in other studies as well? |
| 463 - 466 | Amino acid substitutions within peptides therefore affect peptide recognition by both the TCR and HLA molecules. These complex interactions may be best delineated using bioinformatics approaches, which are beyond the scope of the current experimental analysis presented in this paper. | I understand that it is beyond the scope of this paper, but without this analysis one can only speculate about the final findings and what it means to the research problem. |
| 467 - 470 | Overall, while some amino acid substitutions abrogated the IFN-γ induction potential of immunodominant peptides or resulted in positive responses in both allele peptides albeit at different response magnitudes in some subjects, similar substitutions in peptides did not affect response induction in other subjects. | Aside from the low number of subjects which is definitely a shortcoming of this study, don’t you think the type of amino acid substitution (synonymous and non-synonymous) could affect the structure of this peptide and by extension contributes to variability in immune response among the subjects |
| 476 - 479 | Although we demonstrate an effect of some peptide residue substitutions on the induction of T cell responses, there is no clear pattern regarding which residue changes significantly influence T cell recognition since other similar substitutions did not yield the same effects. | Can this be resolved with the use of more study participants? Was the same found in the CSP study? Why or why not? |
| Discussion does not explore what is required of the ifn-y response for a vaccine as was mentioned in the introduction and how these findings compare against that.  How do the results for ama1 compare to csp which was done in previous studies? What is the implication for malaria vaccine design? It would be helpful to include the role of ifn-y in malaria immune response, even if it is just a few lines. | | |
