# Supplementary material for: Comparative analysis of the ex vivo IFN-gamma responses to CD8+ T cell epitopes within allelic forms of PfAMA1 in subjects with natural exposure to malaria
Source: PLoS One. 2021 Sep 10;16(9):e0257219. doi: 10.1371/journal.pone.0257219 (PMC8432784; doi:10.1371/journal.pone.0257219)
Supplement: Attachment — Submitted filename: Responses to reviewers.docx [file pone.0257219.s003.docx]

| ***Reviewer’s comments for the author.*** | | | **Author responses** |
| --- | --- | --- | --- |
| *The study attempt to understand allelic polymorphisms in the malarial antigen apical membrane antigen 1 (AMA1) and the effect on ex vivo T cell-specific IFN-γ responses in a population with lifelong exposure to malaria.*  *The manuscript is well written.*  *The strength of the manuscript is that it speaks to the design of malaria vaccine candidate, a global need and very relevant to today’s malaria science.*  *The weakness of the manuscript is that the results are based on ONLY 7 candidate/individuals study. This sample size is very small and inferences based on such sample size is statistically challenging.*  *These said, there are a number of issues/concerns that I believe would further improve the manuscript if looked into.* | | | We are grateful for the reviewer’s comments and for identifying weaknesses, which when addressed will improve upon the quality of the paper.  Kindly find our point-by-point responses to the reviewer’s comments below. |
|  | ***Background*** |  |  |
| *Line No* | *Extract from manuscript* | *Reviewer’s comment* |  |
| *Line 49 -51*  *51* | *Malaria remains an economically important disease globally as it results in a huge loss in productivity amongst affected populations and requires significant financial inputs for control and prevention.*  *found mostly in Africa region* | *This statement should be ref*  *grammatical errors ? rephrase* | A reference has been provided for this statement  This has been rectified to read  “…., which is found mostly in Africa, ….. |
| *54 - 55* | *available evidence points to the need for additional complementary tools to ensure disease elimination and eradication* | *Vague, what is the evidence? Why is there the need for these complementary tools* | This statement has been clarified in the revised manuscript |
|  | ***Materials and Methods*** |  |  |
| *100 -125*  *101* | *This study therefore investigated the effect of allelic polymorphisms in MHC-restricted peptides from AMA1 on ex-vivo malaria parasite-specific IFN-γ responses in a population with history of* | *The authors should state the rationale for their selection of AMA1 considering there are other protective antigens in P. falciparum that have been confirmed to show huge allelic polymorphism* | We have provided a reason for selecting AMA1 for this work in the manuscript. |
| *116* | ***Study Site, participants and sample collection*** | *A map of this location would add value.* | Unfortunately we have not been able to include this figure |
| *119 - 120* | *periods of rainfall between March and November.* | *Were blood samples collected from the seven subjects during this period of March to November when malaria infection is known to be high in this region? The authors need to be specific here* | Yes, samples were collected in late March. The manuscript has been updated accordingly. |
| *122 - 123* | *participation in a previous study by the same group, in which participants’ HLA A and B types were determined* | *Is this necessary since all participants were males* | This comment is not very clear, but we sought to communicate that, subjects for this study were drawn from subjects from a previous study for whom we already know the HLA types. |
| *125 126* | *All seven subjects met the inclusion criteria and tested negative for malaria parasites by light microscopy.* | *Were the seven subjects randomly selected from different households in Legon community? This should also be stated together with the inclusion criteria to ensure representativeness in this community* | Subjects were selected based on having previously participated in an earlier study where they were HLA typed and were still resident in the study area. This has been clarified in the revised manuscript. |
| *127* | *Sixty ml (60 ml) of venous blood was collected per subject into heparinized tubes* | *Was the isolation of PBMC from the blood done on the same day of sample collection? If no, then the authors should state the procedure for preservation of the blood samples* | PBMCs were processed on the same day of sample collection. This is clearly stated in the manuscript |
| *128* | *previously described* [1] | *Briefly in one or two sentences summarized this procedure.* | Details of the procedure have been provided |
| *135* | *NetMHC algorithm* | *Provide the parameters of NetMHC used for 9-10mer peptide prediction* | Peptides were identified as potentially immunodominant when the NetMHC algorithm makes a prediction that they are high affinity binders, with IC_50_ of 500 nm or less. The manuscript has been updated accordingly with this information. |
| *137* | *was aligned* | *The author should state the kind of alignment and the software used* | These were linear amino acid sequence alignments, performed using an in-house Microsoft Excel-based alignment algorithm |
| *141 - 142* | *have amino acid variability and, based on the multiple sequence alignments, were selected* | *Sentence structure needs revision* | The sentenced has been revised for clarity |
| *145* | *and following alignment with the other four allelic AMA1 sequences* | *What tool was used for this alignment??* | Tool used for sequence alignment has already been provided in response to a comment above (Line 137) |
| *146 - 147* | *an additional 49 peptides with variability in at least one amino acid residue position relative to the predicted 3D7 consensus peptides were identified.* | *This speaks of the result of this alignment and as such should be in the result section* | The said text has been moved from the methods section to the results section |
| *149 - 151* | *Thirty-five (35) of the 42 predicted 3D7 peptides were shared with one or more of the 4 other parasite strains, while the seven remaining peptides were unique to the 3D7 clone only* | *All these should be in the results section* | The said text has also been moved to the results section |
| *152 - 153* | *All synthesized peptides had > 91 % purity and were originally lyophilized. Peptides were initially dissolved in DMSO and diluted with sterile plain RPMI-1640 to a stock concentration of 10 mg/ml.* | *Peptide synthesis does not fall under the sub-heading “****Predicted 3D7 class 1 epitopes and matching variant sequence synthesis”.*** *As such it should be under a new sub-heading and the procedure for synthesis should be stated.* | The sub-sections have been separated, and additional details on peptide synthesis procedure have been added. |
| *187 – 189* | *For peptides within an allelic set, sfc/m were compared by either Student t test (for paired peptides) or by One-way ANOVA (for more than 2 peptides) as* | *Was this done manually or how?? Please give more information* | These comparisons were done using GraphPad Prism. The manuscript has been duly updated |
| ***Results*** | | |  |
| *221* | *to PBMC stimulations by all peptides ranged from 0 to 66 sfc/m.* | *There is high variation in this from one participant to the next. I see it is mentioned in the discussion also, but what is the implication of that on the study and statistical analysis?* | These background responses are subtracted from the observed peptide/stimulant-specific responses before analysis, and so should not affect the results and interpretation as presented. In naturally exposed persons, we do periodically see a few persons with above regular PBMC activation status, and this shows up as the higher backgrounds, but as stated, the results that are compared have these backgrounds (for every subject) subtracted. |
|  | ***Fig 1. Alignment of P. falciparum AMA1 sequences from the five parasite strains.*** | *figure 1- Figure resolution needs improvement* | The *.tiff* image file quality has been enhanced. Because of the inclusion of a map of the study site (Fig. 1). |
| *321* | ***Table 4: Outcome of subject 4 PBMC stimulation with allelic peptide sets*** | *Why is this table separate from Table 3? Combining both might improve presentation.* | Tables and text are intentionally presented separately for these four subjects, to allow for a detailed description of results for each subject. |
| *365* | *that did not elicit positive T cell responses.* | *Is the positive IFN-gamma associated with T-cell response. Please clarify* | The IFN-gamma response is indeed the T cell response we are assessing. This has been clarified in the revised manuscript. |
| *371 -376* | *Of the eight positive peptides whose corresponding alleles showed statistically significantly less responses, a total of 12 amino acid substitutions were present. Three substitutions occurred at each of amino acid positions 1 and 6. Substitutions at position 1 were from a polar to a positively charged residue (Table 1), from a negatively charged to a positively charged residue (Table 2) and from one non-polar residue to another (Table 3). At position 6, substitutions were from polar to a positively charged residue (Table 1) and two from non-polar to a polar residue (Tables 1 and 4).* | *Was a significant relationship observed between the polarity of amino acids and immune induction (In this case IFN-gamma and T-cell)? I think this area should have focus on whether the amino acid substitution result in changes of the peptide structure that could have resultant effect on its ability to induce immune response.* | Although our results show differences in IFN-gamma responses to some allelic peptides, we did not find a definite relationship or trend between amino acid polarity and the induced immune response. Yes, we agree that amino acid substitutions could result in changes in peptide structure that would affect the peptide’s immunogenicity, but the detailed mechanisms of this was beyond the scope of this study. We are now planning studies that will look at the details of amino acid substitutions on peptide immunogenicity. |
| *384 - 385* | ***Fig 2. Frequency of polymorphic residue distribution at each amino acid position within test peptides.*** | *A sharp figure is require. Figure resolution can be improved* | The *.tiff* image file quality has been enhanced. |
| *This study only used 7 subjects, but the results showed variability between those 7. What is expected for larger sample sizes? Would the variability even out? What does this mean for the population at large* | | | As previously mentioned, the 7 subjects were selected to meet two critical criteria; HLA typed persons from our previous study (so we already have their HLA data and would not need to perform that again) and continued residence in the study area.  We expect to see trends of the relationship between substitutions and the induction of immune responses in our next studies involving a bigger sample size based on this initial finding. We have in the past worked on identifying T cell epitopes in smaller numbers of subjects [1–3], just as is presented here, and have used findings from these studies as basis to get a larger grant that allowed us to recruit up to 300 subjects. A paper from this large study is in the process of being published (Kusi et al, under review) |
|  | ***Discussion*** |  |  |
|  |  | *Some questions I thought of during results were addressed here, which is good.* |  |
| *424 - 425* | *Three of the 18 positive peptides had IFN-γ responses above 100 sfc/m PBMCs while those of the other 15 positive peptides were between 10 and 100 sfc/m.* | *As mentioned in the results, the variation may be allowed but does this mean It has no significant effect on the analysis?* | Yes, this has been explained above |
| *450 - 451* | *The predominant positions where changes that accounted for different responses occurred were at residues 1 and 6.* | *Have these residues been reported as being of interest in other studies as well?* | To the best of our knowledge, no study has so far reported experimental data on how substitutions affect HLA class 1 recognition and immune response induction. We found a paper that showed changes in certain residue positions within short peptides from *Streptococcus* either decreased or increased specific T cell clone proliferation following TCR recognition [4]. In that paper, 30-50% of substitutions at positions 1, 3, 4, 6 and 7 affected T cell proliferation, while only 10% of substitutions at positions 2 and 5 affected T cell proliferation. This study did not however assess HLA recognition by the altered peptides. It is also important to note that the reported residues of importance are based on the variant peptides used in this study, and these did not have equal numbers of substitutions at all 9 to 10 amino acid positions. |
| *463 - 466* | *Amino acid substitutions within peptides therefore affect peptide recognition by both the TCR and HLA molecules. These complex interactions may be best delineated using bioinformatics approaches, which are beyond the scope of the current experimental analysis presented in this paper.* | *I understand that it is beyond the scope of this paper, but without this analysis one can only speculate about the final findings and what it means to the research problem.* | Different responses to allelic peptide variants were observed as reported. It is rather the mechanisms underlying these observations that could not be established here, and we have indicated the other interactions that could possibly explain the observations, but their assessment was not within our current study scope.  This is the basis upon which we have drawn our conclusions |
| *467 - 470* | *Overall, while some amino acid substitutions abrogated the IFN-γ induction potential of immunodominant peptides or resulted in positive responses in both allele peptides albeit at different response magnitudes in some subjects, similar substitutions in peptides did not affect response induction in other subjects.* | *Aside from the low number of subjects which is definitely a shortcoming of this study, don’t you think the type of amino acid substitution (synonymous and non-synonymous) could affect the structure of this peptide and by extension contributes to variability in immune response among the subjects* | Yes, changes in peptide structure due to substitutions could affect peptide recognition by both HLA and TCR, but once again, we did not do any structural analysis in this study and can therefore only speculate |
| *476 - 479* | *Although we demonstrate an effect of some peptide residue substitutions on the induction of T cell responses, there is no clear pattern regarding which residue changes significantly influence T cell recognition since other similar substitutions did not yield the same effects.* | *Can this be resolved with the use of more study participants? Was the same found in the CSP study? Why or why not?* | We believe this can rather be resolved with identifying a specific HLA allele/cognate peptide pair, synthesizing different peptide variants with amino acids of different sizes/polarities and assessing how these variant peptides are recognized by the cognate peptide. We have submitted a grant with this idea and hope to test these in as many subjects as we can identify.  This is because the peptide amino acid residues of importance for binding and hence T cell activation seem to vary depending on the HLA and/or TCR types of subjects whose PBMCs are used for this analysis. |
| *Discussion does not explore what is required of the ifn-y response for a vaccine as was mentioned in the introduction and how these findings compare against that.*  *How do the results for ama1 compare to csp which was done in previous studies? What is the implication for malaria vaccine design? It would be helpful to include the role of ifn-y in malaria immune response, even if it is just a few lines.* | | | We have updated the discussion to indicate the impact of our results on the IFN induction if peptides were included in vaccines.  We have however only done these polymorphism analyses for AMA1 and not CSP. Our previously published paper on CSP [3] was aimed at deconvoluting positive CSP peptide pools that we found in a preceding study [1]. We however hope to analyse the effect of polymorphisms in CSP subsequently. |

**References cited**

1. Ganeshan H, Kusi KA, Anum D, Hollingdale MR, Peters B, Kim Y, et al. Measurement of ex vivo ELISpot interferon-gamma recall responses to Plasmodium falciparum AMA1 and CSP in Ghanaian adults with natural exposure to malaria. MalarJ. 2016;15:55.

2. Anum D, Kusi KA, Ganeshan H, Hollingdale MR, Ofori MF, Koram KA, et al. Measuring naturally acquired ex vivo IFN-γ responses to Plasmodium falciparum cell-traversal protein for ookinetes and sporozoites (CelTOS) in Ghanaian adults. Malaria Journal. 2015;14:20.

3. Kusi KA, Aggor FE, Amoah LE, Anum D, Nartey Y, Amoako-Sakyi D, et al. Identification of Plasmodium falciparum circumsporozoite protein-specific CD8+ T cell epitopes in a malaria exposed population. PLOS ONE. 2020;15:e0228177.

4. Chen YZ, Matsushita S, Nishimura Y. Response of a human T cell clone to a large panel of altered peptide ligands carrying single residue substitutions in an antigenic peptide: characterization and frequencies of TCR agonism and TCR antagonism with or without partial activation. J Immunol. 1996;157:3783–90.
