# Supplementary material for: Comparative analysis of the ex vivo IFN-gamma responses to CD8+ T cell epitopes within allelic forms of PfAMA1 in subjects with natural exposure to malaria
Source: PLoS One. 2021 Sep 10;16(9):e0257219. doi: 10.1371/journal.pone.0257219 (PMC8432784; doi:10.1371/journal.pone.0257219)
Supplement: Attachment — Submitted filename: Responses to reviewer comments.docx [file pone.0257219.s004.docx]

**Responses to Reviewer comments**

Dear Editor,

We are grateful once again for the reviewer’s comments which will ultimately help improve upon the manuscript. Kindly find our point-by-point responses to the comments below.

Reviewer #2: In this study, the authors analyse the effect of polymorphisms affecting CD8+ T cell epitopes from PfAMA-1 sequences on IFNgamma responses measured on PBMCc from 7 subjects living in malaria endemic regions. Those polymorphisms represent PfAMA-1 allelic sequences from 3D7, 7G8, FVO, FC27 and tm284 parasite strains. The authors state that the aminoacidic substitutions do not show a pattern of association with response abrogation.

Main comments:

-Although the authors explain the limitations of their conclusions regarding specifically the (very) low number of subjects included in their study, they extrapolate anyway their findings to a “malaria exposed population” in the title, abstract (line 30) and introduction (line 102). In the opinion of this reviewer, the less assertive term used in the discussion “subjects naturally exposed to malaria” (line 438) is more in agreement with the study, as 7 subjects are hardly representative of a population.

Response
We agree with the reviewer on this point, and have accordingly revised the title to read as follows:

**Comparative analysis of the *ex vivo* IFN-Gamma Responses to CD8+ T cell epitopes within allelic forms of *Pf*AMA1 in Subjects with Natural Exposure to Malaria**

We have also edited the relevant portions of the manuscript to reflect this

-The main concern I found is related to statistical and data analysis mentioned in Materials and Methods section (lines 189-201) and the data showed in the Tables 1-4. The authors state a positivity criteria for IFNgamma response as “at least a doubling of sfc/m in test wells relative to controls (…) in addition to a difference of at least ten spots between test and control wells”. Ten spots in wells containing 400,000 cells/well correspond to 25 sfc/m (million); in that case, 25 sfc/m should be the minimum to consider the sample positive, in the case of a background of zero spots. As in the tables 1-4, there are samples considered positive with as low as 10 sfc/m, either the positivity criteria are wrongly stated, or the analysis is incorrect, or something is missing. From the line 459 in Discussion “responses below 10 sfc/m PBMCs over background responses” I gather that the sfc/m described in the tables are the values after subtraction of background. If that if the case, this is not clear for the reader and should be stated in the MM section and in the legends of the tables. Also, it is not clear if these criteria are applied to each replicate or to averages, please clarify.

Response
The positivity criteria as defined is based on the spot forming cells per million (sfc/m) PBMCs and not on the 400,000 PBMCs used in the assays. The sfc/m value is considered standardized for across study comparisons, since other studies could plate different numbers of PBMCs. These same criteria and approach have been applied in our earlier studies [1–3]. We have however further clarified this in the data analysis section of the manuscript.

The data presented in Tables 1 to 4 are indeed the sfc/m after background subtraction. All reads, including positive controls, test stimulants and background, were first converted to sfc/m before background subtraction for test stimulants and positive controls. These were stated in the statistical analysis section and have been better clarified now. We have also included additional footnotes to Tables 1 to 4 to make these clearer as requested.

It is also confusing that the averages of sfc/m, after all calculations (average of triplicates and extrapolation from 400,000 cells to 1,000,000) are mentioned as whole numbers in the tables. This apparently substantial approximation should be mentioned.

Response

This was deliberate as we are dealing with cells and cannot be counting fractions of cells. The numbers have been rounded off to the nearest whole number, and we do not believe that this should have a significant effect on the data as presented. We have however indicated that these numbers are rounded to the nearest whole number in the table footnote, at least for Table 1.

It should be interesting to know if the stimulations which needed to exclude one replicate (13%) corresponded to one particular subject, as this could be related or not to the high background mentioned for some subjects.

Response

These are single replicate spot readings that looked highly different from the remaining two duplicate readings in 13 % of stimulations, with no more than two such cases per subject. It is therefore not related to observations for any particular subject.

Taking into account all these concerns, in my opinion the raw data of spots/well or at least sfc/m ± SD corresponding to the samples mentioned in the tables (both control and stimulated) should be presented in a supplementary table as supporting information.

Response

We have presented the sfc/m values (after background subtraction) as well as the unstimulated background responses for each subject as a footnote to their respective data tables, so this should allow readers to estimate the sfc/m before background subtraction.

Once again, we are grateful to the reviewer for pointing out these deficiencies and to the editor for allowing us to address these concerns and re-submit our manuscript.

**References**

1. Anum D, Kusi KA, Ganeshan H, Hollingdale MR, Ofori MF, Koram KA, et al. Measuring naturally acquired ex vivo IFN-γ responses to Plasmodium falciparum cell-traversal protein for ookinetes and sporozoites (CelTOS) in Ghanaian adults. Malaria Journal. 2015;14:20.

2. Ganeshan H, Kusi KA, Anum D, Hollingdale MR, Peters B, Kim Y, et al. Measurement of ex vivo ELISpot interferon-gamma recall responses to Plasmodium falciparum AMA1 and CSP in Ghanaian adults with natural exposure to malaria. MalarJ. 2016;15:55.

3. Kusi KA, Aggor FE, Amoah LE, Anum D, Nartey Y, Amoako-Sakyi D, et al. Identification of Plasmodium falciparum circumsporozoite protein-specific CD8+ T cell epitopes in a malaria exposed population. PLOS ONE. 2020;15:e0228177.
